# Helicobacter pylori infection: old and new

**Published:** 2017

**Authors:** S Diaconu, A Predescu, A Moldoveanu, CS Pop, C Fierbințeanu-Braticevici

**Affiliations:** * Department of Internal Medicine II and Gastroenterology, University Emergency Hospital, Bucharest, Romania; ** “Carol Davila” University of Medicine and Pharmacy, Bucharest, Romania

**Keywords:** Helicobacter pylori, gastritis, ulcer, gastric carcinoma, proton pump inhibitor

## Abstract

Helicobacter pylori is a spiral-shaped bacterium that grows in the digestive tract and may be present in more than half of the world’s population.

The clinical features of Helicobacter pylori range from asymptomatic gastritis to gastrointestinal malignancy. Mucosa-associated lymphoid tissue (MALT) lymphoma is a low-grade B-cell marginal zone lymphoma and Helicobacter pylori has been detected in more than 75% of the patients with MALT lymphoma.

Many tests for the detection of Helicobacter pylori are available, including antibody tests, urea breath tests, stool antigen tests and endoscopic biopsies.

The eradication of Helicobacter pylori usually prevents the return of ulcers and ulcer complications even after appropriate medications such as PPIs are stopped. The eradication of Helicobacter pylori is important in the treatment of the rare condition of the stomach known as MALT lymphoma. The treatment of Helicobacter pylori to prevent stomach cancer is controversial.

Confirmation of eradication is recommended in associated ulcers, persistent dyspepsia despite a test-and-treat approach, MALT lymphoma, and previous treatment for early-stage gastric cancer.

The urea breath test and stool antigen test can be used to confirm the eradication and should be performed at least 4 weeks after the completion of therapy.

Several diseases have been reported to be associated with Helicobacter pylori infection, including hematologic diseases, such as ITP, idiopathic iron deficiency anemia and vitamin B12 deficiency. There is a positive trend in the association between Helicobacter pylori infection and neurodegenerative disorders and new data showed a reduced risk of death due to stroke and lung cancer but an increased risk of preeclampsia in infected women, which requires further investigations.

## Introduction

Helicobacter pylori is a spiral-shaped bacterium that grows in the digestive tract. Helicobacter pylori infection has a very high prevalence [**1**], and may be present in more than half of the world’s population. It infects the stomach during childhood. In developing countries, children are very commonly infected. Helicobacter pylori may be passed from person to person through direct contact with saliva, vomit, or fecal matter. Risk factors for Helicobacter pylori infection are related to: living in crowded conditions - living in a home with many people, living without a reliable supply of clean water, living in a developing country, living with someone who has a Helicobacter pylori infection.

The frequency of people infected may somehow be related to race. About 60% of Hispanics, about 54% of African Americans and about 20 to 29% of White Americans have detectable organisms.

Helicobacter pylori is adapted to live in the harsh, acidic environment of the stomach. These bacteria can change their environment and reduce their acidity thus allowing them to survive. While infections typically do not have symptoms, they can lead to other diseases, including peptic ulcers (about 10% of the people infected with Helicobacter pylori) and gastritis. The long-term use of non-steroidal anti-inflammatory drugs also increases the risk of peptic ulcers. Helicobacter pylori gastritis causes a mixed acute and chronic inflammatory reaction, stimulating both neutrophils and eosinophils, as well as mast and dendritic cells. While Helicobacter pylori has been traditionally considered a non-invasive pathogen, recent studies have shown that it is a facultative intracellular bacterium of innate immune cells, capable of interfering with the phagosome maturation which could explain the difficulty in eradicating the bacteria. Helicobacter pylori infection, along with Ebstein-Barr infection, are known risk factors for gastric carcinoma [**2**]. 

**Clinical features**

The clinical features of Helicobacter pylori range from asymptomatic gastritis to gastrointestinal malignancy. An antral-based gastritis occurs in up to 95% of the infected patients and predisposes to duodenal ulcers, whereas the less common corpus-predominant gastritis is a risk factor for gastric ulcers. Up to 50% of the gastric ulcers and 80% of the duodenal ulcers are associated with this infection and the eradication of the organism significantly reduces the risk of ulcer recurrence [**3**]. 

The most common manifestation of Helicobacter pylori is gastritis. Immediately after infection, the bacteria will cause an acute form of gastritis, characterized by hypochlorydia, which will later evolve into a chronic active gastritis that can affect either the antrum (associated with increased acid secretion and duodenal ulcers), the corpus (associated with gastric atrophy and achlorhydria) or both [**4**]. 

**Fig. 1:**
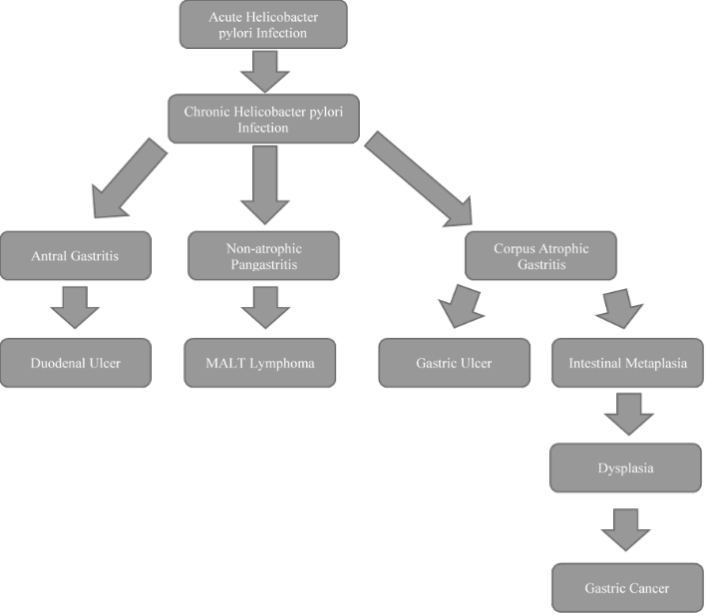
Natural history of infection with Helicobacter pylori

Helicobacter pylori is a group I carcinogen. Mucosa-associated lymphoid tissue (MALT) lymphoma is a low-grade B-cell marginal zone lymphoma and Helicobacter pylori has been detected in more than 75% of the patients with MALT lymphoma. Patients with an early-stage disease are most likely to have a complete remission with the antibacterial treatment and those with a more extensive disease (ulcerations, nodular submucosal mass lesions, invasion throughout the wall or lymphadenopathy) are more likely to require standard lymphoma therapy [**3**]. Patients with intestinal metaplasia (on routine biopsy) should be tested and treated for infection with Helicobacter pylori because intestinal metaplasia is an independent risk factor for gastric malignancy. After the eradication of the organism, the extent to which there is a regression of metaplasia is not currently known. Gastric adenocarcinoma from Helicobacter pylori infection develops through a sequence of gastritis → atrophy → intestinal metaplasia → dysplasia → carcinoma (**Fig. 1**) [**3**]. Patients who undergo endoscopic resection for an early-stage gastric cancer with remaining gastric mucosa should be tested and eradicated because of the risk of metachronous neoplasia.

The most studies showed a higher cumulative incidence of gastric cancer in countries with a higher prevalence of infection. Helicobacter pylori was associated with an increased risk of both diffuse and intestinal type of gastric cancer. Cag-seropositivity was associated with a higher risk of gastric cancer. A recent meta-analysis showed a significant reduction in the risk of gastric cancer in Helicobacter pylori infected subjects who received eradication therapy. Based on these evidences, it was well agreed that Helicobacter pylori is an important risk factor of gastric cancer. However, large well-designed randomized trials are highly anticipated to assess the effectiveness and risks of the “screen and treat” strategy in the development of gastroesophageal reflux disease, obesity and allergic diseases after Helicobacter pylori eradication [**5**]. 

Between 20% and 60% of the patients with functional dyspepsia have an evidence of Helicobacter pylori gastritis and the eradication of the organism results in a symptomatic benefit in a small number of these patients - 10%. Patients younger than 55 years old who have new-onset dyspepsia without alarm features (family history of proximal gastrointestinal cancer, gastrointestinal bleeding, odynophagia, progressive dysphagia, unexplained iron-deficiency anemia, unintentional weight loss, persistent vomiting, palpable mass or lymphadenopathy, jaundice) should undergo Helicobacter pylori testing and treatment if the infection is confirmed. Another option in uninvestigated dyspepsia (in the absence of alarm symptoms) is the use of acid suppression therapy with monitoring for a clinical response.

The relationship of Helicobacter pylori infection and Gastrointestinal Reflux Disease (GERD) has been a long-standing concern. However, the current evidence does not allow definitive conclusions on whether patients with Helicobacter pylori infection have more, less, or equal rates of GERD-related symptoms in comparison with uninfected patients. Studies have not supported the conjuncture that treating Helicobacter pylori infection will worsen GERD.

Helicobacter pylori may represent the main, but not the only, microbial trigger for different gastric diseases and that microorganisms, other than Helicobacter pylori, may play a relevant role in the development of complications in Helicobacter pylori – related gastritis [**6**]. Many bacterial communities, from Prevotella to Streptococcus, have been identified in atrophic gastritis.

A study which was undergone by Del Zompo et al. showed a significant association between the Helicobacter pylori infection and methane production, suggesting the influence gut microbiota composition [**7**]. Enko D and Kriegshauser G consider Helicobacter pylori infection to be significantly associated with the presence of small intestinal bacterial overgrowth - SIBO (determined by functional breath testing). SIBO rates appeared to have increased after eradication therapies [**8**]. The impact of the presence of Helicobacter pylori infection on the reduction of the ferritin and iron levels of coronary artery disease patients as a risk factor independent of other classic factors (including lipid profiles and inflammatory factor) is proved in another study [**9**]. 

During the past year, many articles regarding the extragastric diseases related to Helicobacter pylori infection have been published. The role of Helicobacter pylori in idiopathic thrombocytopenic purpura, iron deficiency anemia, vitamin B12 deficiency is well known; there is a growing interest in the bacterium’s association with cardiovascular, neurologic, hematologic, dermatologic, head and neck, uro-gynecologic diseases, diabetes mellitus and metabolic syndrome with very promising results [**10**]. In a case-control study, the authors observed type 1 diabetic children are at risk of acquiring Helicobacter pylori infection, especially cases with the longer duration of diabetes [**11**]. Recently, an association between Helicobacter pylori infection and preeclampsia was established, suggesting a role for infection in impairing placental development and increasing the risk of developing preeclampsia. The study opens the new perspective of a potential screening and treatment for Helicobacter pylori infection in pregnancy [**12**]. In a randomized, double-blind, placebo-controlled trial it was demonstrated that Helicobacter pylori eradication improves glucose homeostasis in patients with type 2 diabetes (decrease in pro-inflammatory factors) [**13**]. 

Current evidence indicates that Helicobacter pylori eradication therapy, added to iron therapy, might be beneficial in increasing ferritin and hemoglobin levels [**14**]. In a meta-analysis, Upala et al. showed that the infection is positively associated with the metabolic syndrome. Infection with Helicobacter pylori is also associated with higher triglyceride, body mass index, homeostatic model assessment of insulin resistance (HOMA-IR), systolic blood pressure and lower HDL [**15**]. A study which evaluated Helicobacter pylori infection in 58 patients with eosinophilic esophagitis and 116 age and sex-matched controls demonstrated that the Helicobacter pylori infection is inversely associated with eosinophilic esophagitis [**16**]. 

**Diagnostic tests**

Diagnostic tests are indicated in patients:

1) With active peptic ulcer disease (duodenal or gastric),

2) With a history of peptic ulcer disease, who have not been previously treated,

3) With low-grade gastric MALT lymphoma,

4) Who have undergone endoscopic resection of early gastric cancer,

5) With uninvestigated dyspepsia, younger than 55 years old (without alarm symptoms).

Available tests for the detection of Helicobacter pylori include:

- antibody tests,

- urea breath tests,

- stool antigen tests,

- endoscopic biopsies.

Blood tests detect the presence of antibodies of Helicobacter pylori. However, blood antibodies can persist years after the complete eradication of the bacteria. They may be useful in diagnosing the infection, but they are not good for a determined successful eradication.

The urea breath test (UBT) is a safe, easy, and accurate test for the detection of the presence of Helicobacter pylori in the stomach. Ten to twenty minutes after swallowing a capsule containing urea, a breath sample is collected and analyzed for labeled carbon dioxide breath. A positive test signifies that there is an active infection. The test becomes negative shortly after eradication. Individuals concerned with the minute amounts of radioactivity can be tested with urea labeled with heavy, nonradioactive carbon.

Endoscopy is an accurate test for diagnosing the infection as well as the inflammation and ulcers. Endoscopy also allows the determination of the severity of gastritis with biopsies as well as the presence of ulcers, MALT lymphoma and cancer. Biopsies may also be cultured in the bacteriology laboratory for the presence of Helicobacter pylori.

Stool sample: the test uses an antibody of Helicobacter pylori to determine if Helicobacter pylori antigen is present in the stool, which means that there is a Helicobacter pylori infection in the stomach. The stool test can be used to determine if the eradication has been effective after the treatment. In 2012, the FDA approved that the urea breath test was performed in children aged 3 to 17 years.

**Table 1:** Commonly used tests for the detection of Helicobacter pylori infection [**17**,**18**]

TEST	SENSITIVITY	SPECIFICITY
Non-endoscopic tests		
Antibody test (serum)	88-94%	74-88%
Antibody test (whole blood)	67-85%	75-91%
Enzyme-linked immunosorbent assay (serum)	86-94%	78-95%
Stool antigen test	94%	92%
Urea breath test	90-96%	88-98%
Endoscopic tests		
Urease test (biopsy)	88-95%	95-100%
Histology	93-96%	98-99%
Culture	80-98%	100%
Polymerase chain reaction (PCR)	> 95%	> 95%

The sensitivity of UBT is similar to that of rapid urease test (from biopsy) and is reduced by medications that affect urease production; therefore, proton pump inhibitor (PPI) therapy, antibiotic and bismuth therapy should be held for the intervals previously noted.

While more expensive and time consuming than the previous tests, histopathological tests can provide more information related to the consequences of the chronic Helicobacter pylori infection, identifying intestinal metaplasia as well as gastric carcinoma. The recognition of the bacteria has been done routinely by using the hematoxylin and eosin staining, which is appropriate for most cases. The Giemsa staining is also frequently used to diagnose the Helicobacter pylori infection, and has better sensitivity and overall accuracy compared to hematoxylin-eosin [**17**,**19**,**20**]. The histopathological grading as well as the identification of more severe strains, like the cagA-positive strains which have been proven to cause more severe forms of gastritis, could help identify cases at higher risk and provide a guide for the targeted agents [**2**,**21**]. 

PCR test is not widely available or standardized and is not practical for routine diagnosis.

Zeng et al. studied 2199 vaccinated children (with 3 doses of a fusion protein) versus 2204 children on placebo and demonstrated a 55% reduction in the new infections after 2 years [**22**]. 

## Treatment

**Fig. 2:**
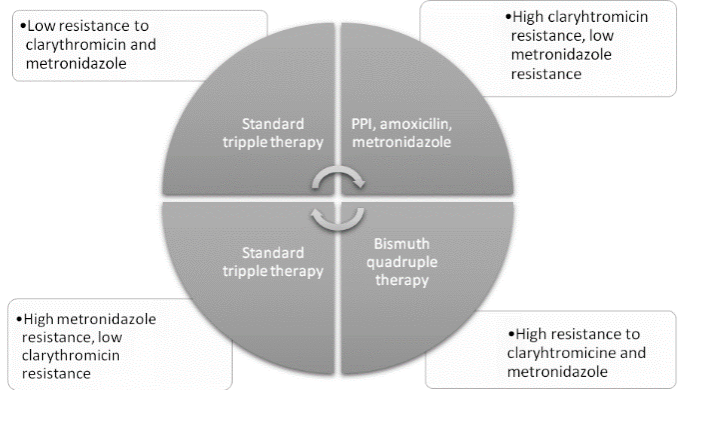
Algorithm for first-line treatment [**3**,**6**]

The eradication of Helicobacter pylori usually prevents the recurrence of ulcers and ulcer complications even after appropriate medications such as PPIs are stopped. The eradication of Helicobacter pylori is important in the treatment of the conditions of the stomach, like MALT lymphoma. The treatment of Helicobacter pylori to prevent stomach cancer is controversial. Standard treatment schemes are detailed in **Table 2**. The treatment for Helicobacter pylori should be offered to all patients with a positive Helicobacter pylori test [**23**]. Helicobacter pylori is difficult to eradicate because it is capable of developing resistance. Therefore, two or more antibiotics are usually given together with a PPI and/ or bismuth containing compounds to eradicate. Studies have shown that the resistance of Helicobacter pylori to clarithromycin is common among patients who have a prior exposure to clarithromycin or other chemically similar macrolide antibiotics (such as erythromycin). The Helicobacter pylori resistance to metronidazole is common among patients who have had a prior exposure to metronidazole. Recommended therapies in areas with antibiotic resistance are detailed in **Fig. 2**. The triple therapy is the most commonly used initial treatment and consists of a PPI, amoxicillin, and clarithromycin. Metronidazole is used in place of clarithromycin if the patient is from an area with an increased clarithromycin resistance, same as bismuth-based regimens which also have the advantage of being low cost. 

**Table 2:** First-line treatment for Helicobacter pylori infection

TREATMENT	DURATION (DAYS)	ERADICATION RATES (%)
PPI, bid Clarithromycin 500 mg, bid Amoxicillin 1000 mg, bid	10-14	70-85
PPI, bid Clarithromycin 500 mg, bid Metronidazole 500 mg, bid	10-14	70-85
PPI, bid Bismuth subsalicylate 525 mg, qid Metronidazole 250 mg, qid Tetracycline 500 mg, qid	10-14	75-90
Sequential Therapy PPI, bid Amoxicillin 1000 mg, bid	5	> 90
followed by: PPI, bid Clarithromycin 500 mg, bid Tinidazole 500 mg, bid	5	
PPI – proton pump inhibitor		

Bacterial antibiotic resistance is regionally variable. Clarithromycin resistance has been rapidly increasing in many countries over the past decade, with rates as high as approximately 30% in Japan and Italy, 40% in Turkey and 50% in China. Resistance rates are much lower in Sweden and Taiwan, at approximately 15%; there are limited data in the USA [**24**]. The sequential therapy has shown promising results, with eradication rates of more than 90%. The treatment failure is usually the result of noncompliance with the medical therapy or of antimicrobial resistance and given the low reinfection rate (< 2% patients for one year). If both clarithromycin and metronidazole resistance is suspected, salvage therapy should be considered. Salvage therapy may be better tolerated and more effective than bismuth-based quadruple therapy, but further validation is required.

The use of high dose PPI twice daily increases the efficacy of triple therapy. Esomeprazole and rabeprazole may be preferred in Europe and North America, where the prevalence of PPI extensive metabolizers is high [**6**]. 

**Treatment strategies for the patient who fails the initial Helicobacter pylori therapy**

Recurrence (reinfection or recrudesces) of the Helicobacter pylori infection after the eradication therapy is also an important issue. Reinfection is defined as infection by a new Helicobacter pylori strain after the confirmation of a successful eradication. Recrudescence is defined as the reactivation of the same strains which became undetected after eradication therapy. Yet, reinfection and recrudesces could not be differentiated in most of the epidemiological studies.

**Fig. 3:**
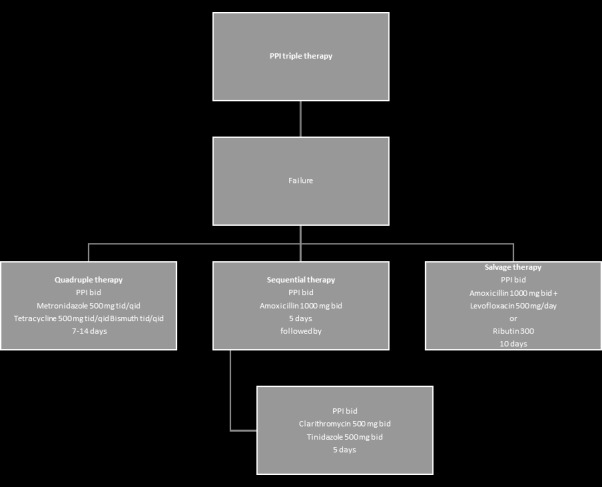
Algorithm for second-line treatment [**6**]

A large randomized controlled intervention trial to prevent gastric cancer by the eradication of Helicobacter pylori in China included 184 786 residents aged 25-54 years, Helicobacter pylori prevalence was 57.6% and the overall eradication rate was 72.9% in the active group. The findings of this trial lead to a distinct evaluation of the factors influencing eradication [**25**]. 

Helicobacter pylori “screen-and-treat” strategies are recommended in communities at high risk of gastric cancer [**6**]. Second line therapies are detailed in **Fig. 3**.

Confirmation of the eradication is recommended in these situations:

- Helicobacter pylori–associated ulcer,

- Persistent dyspepsia despite a test-and-treat approach,

- Helicobacter pylori-associated MALT lymphoma,

- Previous treatment for early-stage gastric cancer.

The urea breath test and stool antigen test can be used to confirm the eradication and should be performed at least 4 weeks after the completion of therapy.

Some authors recommend testing the eradication of infection after treatment in all patients in order to monitor the changes in antibiotic resistance of the bacteria [**26**]. 

**Helicobacter pylori-the truth of today may be the error of tomorrow?**

Over the last year, several diseases have been reported to be associated with Helicobacter pylori infection. In some hematologic diseases, such as ITP, idiopathic iron deficiency anemia and vitamin B12 deficiency, their role has been fully validated and included in the current guidelines. There is a positive trend in the association between Helicobacter pylori infection and neurodegenerative disorders and there is new data concerning a reduced risk of death due to stroke and lung cancer in these patients, but also an increased risk of preeclampsia in infected women, which requires further investigations [**27**].

**Disclosure statement**

The authors declare that there is no conflict of interest regarding the publication of this paper.
